# Synchronous Coexistence of JAK2 V617F‐Positive Essential Thrombocythemia and IgG Kappa Multiple Myeloma in an Octogenarian Patient: A Case Report

**DOI:** 10.1155/carm/8986847

**Published:** 2026-08-26

**Authors:** Marcel Ribero-Vargas, Fabián Ahumada-Córdoba, Nhora María Silva-Pérez, Daniel Ribero-Vargas

**Affiliations:** ^1^ Fellowship of Hematology and Clinic Oncology, Fundación Valle del Lili-ICESI, Cali, Colombia; ^2^ Hematology and Clinic Oncology, Fundación Valle del Lili, ICESI University, Cali, Colombia, icesi.edu.co; ^3^ Pathology and Laboratory Department, Fundación Valle del Lili, Cali, Colombia, valledellili.org; ^4^ Fellowship of Hematology, Universidad de Antioquia, Medellín, Colombia, udea.edu.co

**Keywords:** case report, essential thrombocythemia, JAK2 V617F, multiple myeloma, myeloproliferative neoplasm

## Abstract

The synchronous coexistence of JAK2 V617F‐positive essential thrombocythemia (ET) and IgG kappa multiple myeloma (MM) is exceptionally rare, with fewer than 10 synchronous cases reported in the literature. The present case is unique in that MM was discovered incidentally during systematic bone marrow evaluation for isolated thrombocytosis in an octogenarian with significant comorbidities, requiring the concurrent management of two independent clonal hematological neoplasms. An 81‐year‐old woman with prior hypertension and cerebrovascular disease was referred for persistent thrombocytosis exceeding 1,000,000 platelets/μL. Systematic bone marrow evaluation led to the incidental identification of a monoclonal kappa plasma cell population, prompting full myeloma workup. JAK2 V617F‐positive high‐risk ET and IgG kappa MM (ISS Stage II) were diagnosed synchronously. Acquired von Willebrand syndrome was excluded prior to any anticoagulation decision. Treatment included hydroxyurea—subsequently switched to anagrelide due to a national drug supply shortage—for ET cytoreduction, and a bortezomib–dexamethasone–lenalidomide (VRD) regimen for MM. An IMWG partial response was achieved after three cycles, with adequate platelet control at 8 months of follow‐up. This case underscores the importance of systematic bone marrow evaluation in myeloproliferative neoplasms, as it may unmask concurrent plasma cell dyscrasias. Several pathophysiological mechanisms—including IL‐6‐mediated microenvironmental activation, BAFF‐driven B‐cell proliferation, and a shared inflammatory niche—have been proposed in the literature to explain this co‐occurrence; however, these were not directly evaluated in this patient. The regimen chosen was guided by clinical complexity and resource constraints rather than current preferred first‐line guidelines, which favor daratumumab‐based combinations for transplant‐ineligible patients. Early identification of coexisting clonal neoplasms substantially impacts prognosis and therapeutic decision‐making.

## 1. Introduction

The coexistence of BCR‐ABL1‐negative myeloproliferative neoplasms (MPNs)—including essential thrombocythemia (ET)—with plasma cell dyscrasias such as multiple myeloma (MM) is documented but uncommon, leaving unresolved pathogenic and therapeutic questions [1, 2]. While no standard definition exists in the literature, based on previous reports, for the purposes of this report, synchronous coexistence is defined as the identification of both entities within the same diagnostic workup or within 6 months of each other [3, 4].

The case reported here is of particular interest for several reasons. First, MM was discovered incidentally during systematic workup for isolated thrombocytosis, illustrating the diagnostic value of comprehensive bone marrow evaluation in MPN. Second, the patient was an octogenarian with significant comorbidities—including prior cerebrovascular disease—necessitating simultaneous cytoreductive and antimyeloma therapy, a therapeutic scenario with scarce evidence‐based guidance [5]. Third, this case contributes to the limited body of evidence on independently arising clonal neoplasms [2] and highlights the practical challenges of managing overlapping thrombotic risk [6, 7], acquired von Willebrand syndrome (AvWS) [8], and myelosuppressive toxicities [4].

## 2. Case Report

We present an 81‐year‐old woman with a history of arterial hypertension and ischemic cerebrovascular disease associated with carotid disease four years prior, referred to hematology for persistent thrombocytosis exceeding 1,000,000 platelets/μL over the preceding two years, without leukocytosis or anemia. No family history of hematological malignancy was identified. The patient was functionally independent prior to diagnosis (ECOG performance status 1). No prior hematological interventions or systemic treatments were documented. Patient data have been de‐identified, and written informed consent for publication was obtained from the patient’s legal representative (see Informed Consent section). The patient reported intermittent bone pain and ecchymoses on the lower extremities.

Physical examination was notable for ecchymoses on both lower extremities. No hepatosplenomegaly, lymphadenopathy, or signs of spinal cord compromise were identified. Performance status was ECOG 1.

**FIGURE 1:**
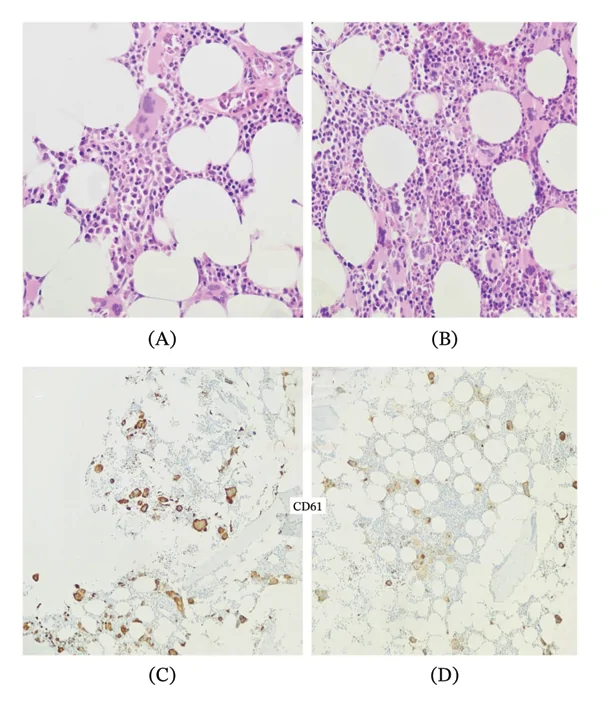
Bone marrow biopsy findings consistent with essential thrombocythemia (JAK2 V617F‐positive). (A, B) Hematoxylin and eosin (H&E) staining (× 400) demonstrating normocellular bone marrow with prominent megakaryocytic hyperplasia. No significant reticulin fibrosis, dysplastic features, or blast excess is identified. (C, D) Immunohistochemistry for CD61 (Platelet glycoprotein IIIa; × 100) confirming megakaryocytic lineage. CD61‐positive megakaryocytes are diffusely distributed throughout the biopsy, with focal clustering, consistent with the diagnosis of essential thrombocythemia.

Initial bone marrow biopsy revealed normocellular marrow with megakaryocytic hyperplasia without fibrosis or dysplasia (Figures 1A–D). JAK2 V617F mutation was confirmed, establishing the diagnosis of high‐risk ET. Cytoreductive therapy was initiated with hydroxyurea and low‐dose acetylsalicylic acid.

Prior to initiating antiplatelet therapy, AvWS was evaluated given the platelet count exceeding 1,000,000/μL. The vWF ristocetin cofactor/antigen ratio was within normal limits, ruling out clinically significant AvWS at the time of assessment.

**FIGURE 2:**
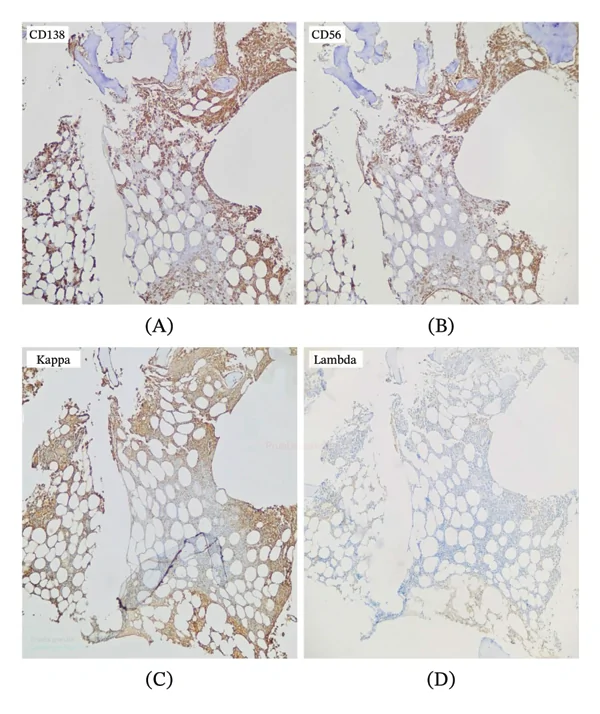
Bone marrow biopsy immunohistochemistry confirming plasma cell myeloma with aberrant phenotype and kappa light chain restriction (× 40). (A) CD138 immunostaining showing extensive diffuse positivity, identifying a markedly expanded plasma cell population occupying approximately 60% of the bone marrow space. (B) CD56 immunostaining demonstrating co‐expression in the neoplastic plasma cell population, an aberrant finding not seen in normal plasma cells, consistent with myelomatous infiltration. (C) Kappa light chain immunostaining revealing strong, diffuse cytoplasmic positivity throughout the infiltrating plasma cell population. (D) Lambda light chain immunostaining showing absent staining in the plasma cell population, confirming kappa light chain restriction and monoclonality. Together, Panels A–D establish the diagnosis of IgG kappa multiple myeloma with aberrant CD56 expression.

Bone marrow aspirate flow cytometry identified 4.3% monoclonal plasma cells (CD38+/CD138+/CD56+/CD117+, kappa restriction), prompting a complete myeloma workup. Serum immunofixation revealed an IgG kappa monoclonal band; protein electrophoresis showed an M‐protein of 4.6 g/dL; serum free light chain kappa/lambda ratio was 45; hemoglobin was 9.5 g/dL. PET–CT demonstrated multiple hypermetabolic lytic bone lesions (Deauville score 5). Bone marrow biopsy confirmed 60% infiltration by neoplastic plasma cells (Figure 2A–D), establishing a diagnosis of symptomatic IgG kappa MM, ISS Stage II (albumin 3.1 g/dL; β2‐microglobulin 3.5 mg/L). FISH‐based cytogenetic risk stratification—including del (17p), t (4; 14), t (14; 16), 1q21 gain, and 1p deletion—was not available at the time of diagnosis; R‐ISS and R2‐ISS staging could therefore not be determined.

Given the patient’s age (81 years), ECOG 1 performance status, prior cerebrovascular event, and the complexity of managing concurrent high‐risk ET, a doublet induction regimen of bortezomib–dexamethasone (VD) was chosen as the initial approach. Once platelet count responded to hydroxyurea (Month 3), lenalidomide was added, completing a VD–lenalidomide (VRD) regimen. The clinical timeline is summarized in Table 1.

**TABLE 1:** Clinical timeline. Chronological sequence of diagnostic and therapeutic events throughout the episode of care.

Timepoint	Events
∼24 months prediagnosis	Persistent thrombocytosis > 1,000,000/μL noted; patient referred to hematology
Month 0—Week 1	Initial hematology evaluation. Bone marrow biopsy: normocellular marrow, megakaryocytic hyperplasia, no fibrosis. JAK2 V617F positive ⟶ ET diagnosis (high‐risk: age > 60, prior stroke, platelets > 1M). Hydroxyurea 500 mg twice daily + low‐dose ASA initiated. Acquired von Willebrand syndrome ruled out: vWF: RCo/vWF: Ag ratio within normal limits
Month 0—Week 3	Bone marrow flow cytometry: 4.3% monoclonal kappa plasma cells (CD38+/CD138+/CD56+/CD117+). Full MM workup initiated
Month 1	MM workup complete: SPEP M‐protein 4.6 g/dL, IFE IgG kappa, FLC *κ*/*λ* ratio 45, Hb 9.5 g/dL, PET‐CT multiple hypermetabolic lytic lesions (Deauville 5), repeat BM biopsy 60% plasma cells. IgG kappa MM ISS II confirmed. VD initiated: bortezomib 1.3 mg/m^2^ SC days 1, 4, 8, 11 + dexamethasone 40 mg weekly. Note: FISH for cytogenetic risk stratification was not performed (see Limitations).
Month 1—Concurrent	Platelet count began declining on hydroxyurea
Month 3	Lenalidomide 25 mg days 1–21/28 added (VRD regimen). Grade 1 peripheral neuropathy noted (bortezomib); no dose reduction. Myeloma response assessment: M‐protein 1.9 g/dL (> 50% reduction from baseline 4.6 g/dL) ⟶ IMWG partial response confirmed. Serum FLC kappa/lambda ratio improved from 45–9.2
Month 3‐4	Hydroxyurea discontinued due to national supply shortage. Anagrelide 0.5 mg twice daily initiated for continued ET cytoreduction
Month 8 (last follow‐up)	Platelets 422,000/μL on anagrelide. Myeloma: best response PR (maintained). No thrombotic or hemorrhagic events. ECOG 1. Patient ambulatory, continuing VRD outpatient

Hydroxyurea was discontinued at Months 3‐4 due to a national drug supply shortage and was substituted with anagrelide 0.5 mg twice daily for continued ET cytoreduction. The platelet count at the time of the last follow‐up visit (Month 8) was 422,000/μL under anagrelide therapy.

Myeloma response was formally assessed per IMWG criteria [9] after three completed VRD cycles: serum M‐protein had decreased from 4.6 to 1.9 g/dL (> 50% reduction), meeting the criteria for partial response (PR). Serum FLC kappa/lambda ratio improved from 45 to 9.2. A repeat bone marrow biopsy for deeper response assessment was not performed given the patient’s age and adequate clinical response. The adverse event profile included Grade 1 peripheral neuropathy attributable to bortezomib, managed conservatively without dose reduction. No significant cytopenias, thromboembolic events, or infections were recorded during the 8‐month follow‐up period.

## 3. Discussion

Ten cases of synchronous MM and ET have been reported in the English‐language literature, with ET predominating as the first diagnosis (Table 2) [4, 10–16].

**TABLE 2:** Published cases of synchronous coexistence of essential thrombocythemia and multiple myeloma.

#	Reference	Age/sex	Driver mutation	M‐Protein	Plt × 10^9^/L	Treatment	Outcome
1	Selroos and van Assendelft [10]	78/M	Unknown^∗^	IgA kappa	1420	Melphalan	Alive at 15 months
2	Selroos and van Assendelft [10]	66/F	Unknown^∗^	IgG kappa	1220	Busulphan	Died of venous thromboembolism
3	Ghosh [11]	80/F	Unknown^∗^	FLC lambda	920	Unknown	Unknown
4	Kuroda et al. [12]	67/F	JAK2 V617F	IgG lambda	1130	Hydroxyurea for ET; no MM treatment (asymptomatic)	Alive at 12 months; MM asymptomatic
5	Holtan et al. [13]	51/M	JAK2 V617F	NR	9000	Autologous HSCT for MM	CR for MM; ET persisted; alive posttransplant
6	Naeem et al. [14]	32/F	JAK2 V617F	IgG lambda	594	VRD‐based regimen (details NR)	Response achieved; short follow‐up
7	Krishnan et al. [4]	86/F	CALR exon 9	IgG lambda	1518	MPV ⟶ Ld ⟶ DVd ⟶ PCd; hydroxyurea for ET	Multiple CRs; progressed to PC leukemia; deceased
8	Hammani et al. [15]	82/M	JAK2 V617F	IgG lambda (SMM)	946	Aspirin + hydroxyurea for ET; SMM surveillance	No myeloma‐defining events at 1 year
9	Zhang et al. [16]	75/M	JAK2 V617F	IgG kappa	671	VCD (no specific ET therapy)	M‐Protein reduced > 75%; platelets normalized on VCD
10	Present case	81/F	JAK2 V617F	IgG kappa	1000	Hydroxyurea ⟶ anagrelide; VD ⟶ VRD	IMWG PR at 8 months; platelets 422 × 10^9^/L on anagrelide

*Note:* Synchronous coexistence defined as identification of both diagnoses within the same diagnostic workup or within 6 months of each other. DVd, daratumumab–bortezomib–dexamethasone; MPV, melphalan–prednisone–bortezomib; VCD, bortezomib–cyclophosphamide–dexamethasone; VD, bortezomib–dexamethasone; VRD, bortezomib–lenalidomide–dexamethasone.

Abbreviations: CR, complete response; ET, essential thrombocythemia; FLC, free light chain; HSCT, hematopoietic stem cell transplant; IMWG, International Myeloma Working Group; Ld, lenalidomide–dexamethasone; MM, multiple myeloma; NR, not reported; PCd, pomalidomide–cyclophosphamide–dexamethasone; PR, partial response; SMM, smoldering multiple myeloma.

^∗^Driver mutation status not reported; cases predated availability of JAK2 V617F testing (2005).

In addition, Brunner and colleagues analyzed second malignancies in patients with BCR‐ABL1‐negative MPNs using the SEER database, finding an incidence rate ratio of 1.55 (95% CI: 1.00–2.32) for MM development compared to the general population [1]. It can be concluded that the association is not strong, but there does appear to be a modestly increased risk of co‐occurrence between MPNs and monoclonal gammopathies.

### 3.1. Proposed Biological Mechanisms

Several mechanisms have been proposed in the published literature as potentially relevant to the co‐occurrence of MPNs and plasma cell dyscrasias. Importantly, none of these pathways was directly measured or confirmed in the present case; they are discussed here as contextual hypotheses to inform future investigations.

Interleukin‐6 (IL‐6) has been proposed as a shared trophic signal, given its established role as a growth factor for MM and its reported elevation in patients with ET [17]. Marta et al. documented increased plasma IL‐6 and soluble receptor levels in ET patients [17]; however, IL‐6 was not measured in the present patient, and a direct causal relationship cannot be inferred.

In patients with JAK2‐ or CALR‐mutated ET, increased B lymphocyte activity has been reported, attributed to elevated membrane‐bound B cell‐activating factor (mBAFF) [18]. Sustained B‐cell activation may theoretically favor clonal plasma cell evolution; however, mBAFF was not evaluated in this case.

Several reports have demonstrated that MPN driver mutations (JAK2/CALR/MPL) and MM‐associated cytogenetic alterations reside in distinct cell populations, supporting the hypothesis of independently arising clones rather than a shared precursor [12]. In the present case, JAK2 V617F testing on sorted plasma cells was not performed, so direct evidence of biclonality is lacking. The independent origin hypothesis remains the most parsimonious explanation but requires molecular confirmation.

### 3.2. Cytogenetic Risk Stratification

A recognized limitation of this case is the absence of a FISH‐based cytogenetic panel for MM risk stratification, precluding R‐ISS or R2‐ISS staging. Current IMWG recommendations mandate plasma cell FISH (including del [17p], t [4; 14], t [14; 16], t [14; 20], 1q21 gain/amplification, and 1p deletion) for all newly diagnosed MM patients, as these findings directly impact prognosis and treatment decisions [9]. This limitation is acknowledged; centers managing similar cases should prioritize cytogenetic workup to enable standard risk stratification.

### 3.3. Therapeutic Considerations

In the absence of specific guidelines for synchronous MPN–MM, the most accepted approach is to prioritize treatment of the entity posing the greatest immediate risk of morbidity and mortality [4, 5]. In our patient, both high‐risk ET (platelet count > 1M, prior stroke) and symptomatic MM (anemia and lytic bone lesions) required concurrent management.

Hydroxyurea was used off‐label in this patient for high‐risk ET in the setting of a concurrent MM diagnosis—a scenario for which long‐term safety data are scarce. The claim of hydroxyurea safety in MM requires important qualification. Sterkers et al. reported that 54% (7/13) of ET patients developing AML/MDS after hydroxyurea exposure carried 17p deletions, a high‐risk cytogenetic abnormality associated with TP53 loss, raising concern about leukemogenic potential in genomically susceptible individuals [19]. The landmark Finazzi et al.’s randomized trial documented a 3.9% secondary malignancy rate with hydroxyurea monotherapy; therefore, the elevated rate of 33% observed with sequential busulphan followed by hydroxyurea reflects alkylator exposure rather than hydroxyurea alone, and no busulphan‐monotherapy arm was included in that study [20]. There is only a report of MM developing 3.5 years after the initiation of anagrelide in a 52‐year‐old male patient with ET; however, a causal relationship is not considered likely. In this case, the authors propose a carcinogenic mechanism independent of cytotoxic effect [21]. In the absence of FISH cytogenetics for del (17p), the leukemogenic risk of hydroxyurea in the present patient cannot be formally excluded. Our recommendation is that clinicians managing similar cases should perform cytogenetic risk stratification before or concurrent with hydroxyurea initiation, consider alternative cytoreductive agents (anagrelide and interferon‐α) if del (17p) is identified or if long‐term safety in combined hematological malignancy is a concern, and explicitly document the off‐label nature of hydroxyurea use in this clinical context.

Regarding the antimyeloma regimen, it is important to acknowledge that the therapeutic approach employed in this case—sequential VD doublet followed by escalation to VRD—does not reflect the current preferred first‐line therapy for transplant‐ineligible MM patients. Current NCCN guidelines [22] designate daratumumab‐based triplet and quadruplet combinations (DRd, DVRd, and Isa‐VRd) as preferred regimens, while VRD is classified as “Other Recommended” (Category 1) and VD alone is listed only as “Useful in Certain Circumstances.” The choice of VD induction in our patient was guided by several clinical factors specific to this case: advanced age (81 years), ECOG 1 performance status, prior cerebrovascular disease, the complexity of concurrent high‐risk ET management, and the deliberate decision to defer early lenalidomide introduction due to concern about compounding thrombotic risk. While the PR achieved supports the clinical utility of VRD in this context, we acknowledge this as a deviation from current standard‐of‐care and encourage clinicians managing similar cases to consider daratumumab‐based combinations when feasible, after appropriate thrombotic and cytoreductive risk assessment.

### 3.4. Thrombosis and Bleeding Risk

Both MM and ET independently confer elevated arterial and venous thrombotic risk [6, 7]. In the context of concurrent disease, thrombosis risk assessment requires integration of tools from both disciplines. The IPSET‐Thrombosis score is the validated instrument for ET‐specific risk stratification and notably does not include platelet count as a variable, as platelet count per se does not correlate with thrombotic risk in ET [7]. The IMPEDE‐VTE score, while useful for MM‐specific VTE prediction, was designed for myeloma patients and does not account for MPN‐related thrombocytosis [6]; applying it in isolation in this setting is therefore insufficient.

Crucially, extreme thrombocytosis (≥ 1000 × 10^9^/L) is associated with paradoxically increased bleeding risk through AvWS, in which large vWF multimers are adsorbed onto the platelet surface and degraded [8]. Clinicians must recognize that recommending anticoagulation without first excluding AvWS in a patient with extreme thrombocytosis risks precipitating serious hemorrhage [8].

### 3.5. Strengths and Limitations

The principal strengths of this case are the systematic diagnostic approach, IMWG‐compliant myeloma staging including PET‐CT and serial M‐protein assessment, formal AvWS evaluation prior to anticoagulation decisions, and the detailed description of therapeutic sequencing under comorbid constraints. Limitations include the following: the retrospective single‐case design; absence of FISH cytogenetics for MM risk stratification, precluding R‐ISS/R2‐ISS staging; lack of JAK2 V617F testing on sorted plasma cells, preventing formal biclonality confirmation; the 8‐month follow‐up duration, which precludes definitive conclusions about long‐term outcomes; and the inability to obtain a formal written patient perspective statement. These limitations do not diminish the clinical educational value of the case but are disclosed in accordance with CARE reporting standards.

This case highlights the importance of maintaining systematic diagnostic vigilance in patients with isolated thrombocytosis, as bone marrow evaluation may reveal concurrent plasma cell dyscrasias with direct therapeutic implications. The synchronous management of high‐risk ET and MM in an elderly patient with comorbidities requires individualized, risk‐stratified decision‐making integrating both MPN and myeloma guidelines. Cytogenetic characterization, AvWS exclusion, and formal myeloma response assessment should be considered essential components of care in this rare clinical scenario.

## Funding

No funding was received for this research.

## Consent

Written informed consent was obtained from the patient’s legal guardian for publication of this case report and accompanying images. A copy of the written consent is available for review by the Editor‐in‐Chief upon request.

## Conflicts of Interest

The authors declare no conflicts of interest.

## Patient Perspective

A formal written patient narrative was not obtained given the patient’s age, health literacy level, and the retrospective nature of this report. The patient verbally communicated satisfaction with the clinical care received, reported adequate pain control, and expressed willingness to continue the prescribed outpatient regimen with good overall tolerability. The absence of a formal written patient statement is acknowledged as a limitation as per the CARE guideline.

## Data Availability

The data that support the findings of this study are available from the corresponding author upon reasonable request.

## References

[1] Brunner A. M. , Hobbs G. , Jalbut M. M. , Neuberg D. S. , and Fathi A. T. , A Population-Based Analysis of Second Malignancies Among Patients With Myeloproliferative Neoplasms in the SEER Database, Leukemia and Lymphoma. (2016) 57, no. 5, 1197–1200, 10.3109/10428194.2015.1071490. 26155828 PMC4943877

[2] Malhotra J. , Kremyanskaya M. , Schorr E. , Hoffman R. , and Mascarenhas J. , Coexistence of Myeloproliferative Neoplasm and Plasma-Cell Dyscrasia, Clinical Lymphoma, Myeloma and Leukemia. (2014) 14, no. 1, 31–36, 10.1016/j.clml.2013.09.015. 24220620

[3] Gao H. , Wang X. , Lai Y. et al., Different Situations of Identifying Second Primary Malignant Tumors in Lymphoma Patients With Synchronous Solid Tumors, Cancer Medicine. (2023) 12, no. 7, 8038–8049, 10.1002/cam4.5592. 36621802 PMC10134266

[4] Krishnan N. , Price R. , and Kotchetkov R. , Case Report: Effects of Multiple Myeloma Therapy on Essential Thrombocythemia and Vice Versa: A Case of Synchronous Dual Hematological Malignancy, Frontiers in Oncology. (2023) 13, 10.3389/fonc.2023.1213942. PMC10287112 37361582

[5] Xiang Q. , Chu B. , Lu M. et al., Coexistence of Myeloproliferative Neoplasms With Multiple Myeloma, Cancer Pathogenesis and Therapy. (2024) 2, no. 3, 212–214, 10.1016/j.cpt.2023.12.001. 39027144 PMC11252990

[6] Frenzel L. , Decaux O. , Macro M. et al., Venous Thromboembolism Prophylaxis and Multiple Myeloma Patients in Real-Life: Results of a Large Survey, Thrombosis Research. (2024) 233, 153–164, 10.1016/j.thromres.2023.11.021. 38064842

[7] Guglielmelli P. , Carobbio A. , Rumi E. et al., Validation of the IPSET Score for Thrombosis in Patients With Prefibrotic Myelofibrosis, Blood Cancer Journal. (2020) 10, no. 2, 10.1038/s41408-020-0289-2. PMC7042364 32098944

[8] Simini G. et al., Thrombocytosis and Bleeding in Myeloproliferative Neoplasms: Exploring Clinical Diversity and Risk of Acquired Von Willebrand Syndrome, Research and Practice in Thrombosis and Haemostasis. (2025) 9, no. 5. 10.1016/j.rpth.2025.102954 PMC12284493 40709222

[9] Kyle R. A. and Rajkumar S. V. , Criteria for Diagnosis, Staging, Risk Stratification and Response Assessment of Multiple Myeloma, Leukemia. (2009) 23, no. 1, 3–9, 10.1038/leu.2008.291. 18971951 PMC2627786

[10] Selroos O. and van Assendelft A. , Thrombocythaemia and Multiple Myeloma. A Report on Two Cases, Acta Medica Scandinavica. (1977) 201, no. 3, 243–247, 10.1111/j.0954-6820.1977.tb15692.x. 557871

[11] Ghosh P. , Essential Thrombocythaemia and Bence Jones Myeloma, British Medical Journal. (1984) 289.

[12] Kuroda J. , Matsumoto Y. , Tanaka R. et al., JAK2V617F-Positive Essential Thrombocythemia and Multiple Myeloma With IGH/CCND1 Gene Translocation Coexist, But Originate From Separate Clones, Acta Haematologica. (2008) 120, no. 3, 177–181, 10.1159/000187645. 19129688

[13] Holtan S. , Hoyer J. , and Buadi F. , Multiple Myeloma With Concomitant JAK2-Positive Essential Thrombocythemia Post-Successful Autologous Peripheral Blood Hematopoietic Stem Cell Transplant, Bone Marrow Transplantation. (2011) 46, no. 4, 10.1038/bmt.2010.157. 20581888

[14] Naeem A. , Amar S. , Mehta D. , and Malik M. N. , Thrombocytosis as an Initial Presentation of Plasma Cell Neoplasm: A Case Report, Cureus. (2019) 11, no. 3, 10.7759/cureus.4286. PMC6538227 31183267

[15] Hammani A. , Doghmi O. , Allaoui M. et al., A Rare Coexistence of Smoldering Multiple Myeloma and JAK2-Positive Myeloproliferative Neoplasm: A Case of Dual Synchronous Hematological Malignancy, Cureus. (2024) 16, no. 1, 10.7759/cureus.52622. PMC10876019 38374866

[16] Zhang F. , Chen Y. , Huang D. , and Chen S. , A Rare Case of Concurrent JAK2V617F-Positive Essential Thrombocythemia, Multiple Myeloma, and Colorectal Adenocarcinoma, Indian Journal of Pathology & Microbiology. (2025) 68, no. 3, 626–629, 10.4103/ijpm.ijpm_990_23. 39773885

[17] Marta R. F. , Goette N. P. , Lev P. R. et al., Increased Levels of Plasma Interleukin-6 Soluble Receptor in Patients With Essential Thrombocythemia, Haematologica. (2004) 89, no. 6, 657–663. 15194532

[18] Lim K. H. , Chen C. G. S. , Chang Y. C. et al., Increased B Cell Activation is Present in JAK2V617F-Mutated, CALR-Mutated and Triple-Negative Essential Thrombocythemia, Oncotarget. (2017) 8, no. 20, 32476–32491, 10.18632/oncotarget.16381. 28415571 PMC5464803

[19] Sterkers Y. , Preudhomme C. , Laï J. L. et al., Acute Myeloid Leukemia and Myelodysplastic Syndromes Following Essential Thrombocythemia Treated With Hydroxyurea: High Proportion of Cases With 17p Deletion, Blood. (1998) 91, no. 2, 616–622, 10.1182/blood.v91.2.616. 9427717

[20] Finazzi G. , Ruggeri M. , Rodeghiero F. , and Barbui T. , Second Malignancies in Patients with Essential Thrombocythaemia Treated With Busulphan and Hydroxyurea: Long-Term Follow-Up of a Randomized Clinical Trial, British Journal of Haematology. (2000) 110, no. 3, 577–583, 10.1046/j.1365-2141.2000.02188.x. 10997967

[21] Leković D. , Gotić M. , Mitrović O. et al., The Emergence of Non-Secretory Multiple Myeloma During the Non-Cytotoxic Treatment of Essential Thrombocythemia, Journal of Medical Case Reports. (2013) 7, no. 1, 10.1186/1752-1947-7-224. PMC3847637 24025541

[22] Kumar S. K. , Callander N. S. , Adekola K. et al., NCCN Guidelines® Insights: Multiple Myeloma, Version 1.2025, Journal of the National Comprehensive Cancer Network. (May 2025) 23, no. 5, 132–140, 10.6004/jnccn.2025.0023. 40340857

